# Moving beyond treatment: pulmonary function, autonomous motivation, and physical activity enjoyment in adults with cystic fibrosis

**DOI:** 10.1186/s12890-026-04661-w

**Published:** 2026-09-07

**Authors:** Wolfgang Gruber, Matthias Welsner, Florian Stehling, Jin-Sun Schermaul, Jose G. Ortiz, Liron Lechtenberg, Christian Taube, Margarete Olivier

**Affiliations:** 1 Paediatric Pulmonology and Sleep Medicine, Cystic Fibrosis Centre, Children´S Hospital, University of Duisburg-Essen, 45147 Essen, Germany; 2 Fachklinik Satteldüne der DRV Nord, Nebel/Amrum, Germany; 3 Department of Pulmonary Medicine, Adult Cystic Fibrosis Center, University Hospital Essen - Ruhrlandklinik, University of Duisburg-Essen, Essen, Germany

**Keywords:** Cystic fibrosis, Self-determination theory, Autonomous motivation, Physical activity enjoyment, Pulmonary function, CFTR modulator therapy, Sex

## Abstract

**Background:**

Physical activity (PA) and structured exercise are established cornerstones of cystic fibrosis (CF) management. While CFTR modulators have transformed treatment, the psychological factors affecting sustained PA adherence, particularly motivational regulation and PA enjoyment, require further investigation. This study aimed to determine whether percent predicted forced expiratory volume in one second (ppFEV₁) is independently associated with autonomous motivation and PA enjoyment in adults with CF, after adjusting for CFTR modulator therapy status and sex.

**Methods:**

A cross-sectional study was conducted at a specialised CF centre (*N* = 200). Participants completed validated questionnaires assessing motivational regulation (Behavioural Regulation Questionnaire-2, BREQ-2) and PA enjoyment (Physical Activity Enjoyment Scale, PACES).

Clinical data, including the ppFEV₁, were obtained from medical records. Multivariate general linear models (GLM), partial correlations, and hierarchical regression analyses were utilised.

**Results:**

Cross-sectional analyses showed that ppFEV₁ was independently associated with intrinsic motivation (β = .233, *p* < .001), the relative autonomy index (RAI) (β = .203, *p* = .004) and the PACES total score (β = .201, *p* = .004). After CFTR modulator therapy status and sex were accounted for, the additional variance explained by the ppFEV₁ was statistically significant but modest (ΔR^2^ = .054, .041, and .040, respectively). Neither the CFTR modulator therapy status nor sex was significantly associated with the assessed motivational or enjoyment outcomes.

**Conclusions:**

Although ppFEV₁ showed small, independent associations with autonomous motivational regulation and PA enjoyment in adults with CF, lung function should not be used as a proxy for motivational support needs. To provide person-centred, SDT-informed support that encourages sustained participation in PA, the motivational regulation and enjoyment of PA should be assessed directly in adults with CF.

**Supplementary Information:**

The online version contains supplementary material available at https://doi.org/10.1186/s12890-026-04661-w.

## Introduction

Cystic fibrosis (CF) is a chronic multisystem disease in which pulmonary impairment remains clinically relevant throughout adulthood [1]. However, contemporary CF care involves more than just pharmacological treatment, and increasingly incorporates methods that improve long-term functioning, participation, and quality of life. Within this broader therapeutic framework, physical activity (PA) and structured exercise are widely considered important components of comprehensive CF care [2].

Although the clinical relevance of PA and exercise in people with cystic fibrosis (pwCF) is well-established, the current evidence requires careful interpretation. A recent Cochrane review reported that PA and exercise interventions with a minimum duration of six months probably increase exercise capacity in pwCF. However, the effects on lung function and health-related quality of life appear to be smaller, inconsistent or uncertain [2].

Moreover, observational data from adults with CF suggest that greater engagement in moderate-to-vigorous PA (MVPA) is linked to more favourable clinical outcomes over time, including increased exercise capacity, higher percent predicted forced expiratory volume in one second (ppFEV_1_) at 12 months, and fewer hospital admissions and days [3, 4]. These findings support PA as a meaningful and potentially modifiable component of long-term CF management, rather than an optional adjunct [2–4].

The therapeutic landscape of CF has changed substantially with the introduction of highly effective cystic fibrosis transmembrane conductance regulator (CFTR) modulator therapy, particularly elexacaftor/tezacaftor/ivacaftor (ETI) [1, 5]. Real-world data has consistently shown improvements in ppFEV₁ and BMI, together with a reduction in the burden of pulmonary exacerbations, following the initiation of ETI [6, 7].

However, whether these physiological gains automatically result in sustained changes in PA behaviour, including structured exercise, or its psychological determinants remain ambiguous [8, 9]. Recent data from studies of adults suggest that ETI treatment may increase habitual PA, including step count, whereas changes in MVPA appear to be less distinct, and the available data remain limited [10]. Other studies have examined changes in walking distance and exercise capacity following treatment with CFTR modulators, as well as the effects of structured exercise interventions in pwCF [10–14]. Therefore, improved ppFEV_1_ and increased exercise capacity alone are unlikely to explain why some adults with CF remain active while others do not.

Barriers to regular PA and structured exercise remain common among adult pwCF. Qualitative research has identified fatigue, breathing difficulties, a lack of available facilities, time constraints, adverse weather conditions, negative perceptions of PA and perceived health risks as barriers to participation in adults with CF. Respiratory benefits, well-being and social support may facilitate participation [15, 16].

A 12-month partially supervised exercise programme found physical and psychological barriers to be more prominent in less active pwCF than in more active individuals and that some barriers are specific to this population. Other CF-specific barriers, such as the risk of cross-contamination and transplant preparation, have also been described [15, 16]. These findings suggest that the central issue is not only whether PA and structured exercise are beneficial in pwCF, but also what motivates adult pwCF to engage in them consistently despite the chronic burden of treatment and disease-related constraints.

This variability in the ability to overcome barriers cannot be explained by disease severity or treatment status only, suggesting that motivational orientation may be an additional moderating factor. Self-determination theory (SDT) provides a useful framework for addressing this issue. SDT distinguishes between different types of motivation, ranging from amotivation and more controlled forms of regulation to more autonomous forms, such as identified and intrinsic regulation [17].

Theoretically, these motivational processes are affected by the satisfaction of three basic psychological needs: autonomy, competence and relatedness [17]. More autonomous motivation is consistently associated with increased participation and sustained PA behaviour [18–20]. Ntoumanis et al. reported that interventions informed by SDT are often effective, with small-to-moderate effects frequently mediated by increases in autonomous motivation, support for interpersonal needs, and satisfaction of competence needs [20]. Similarly, Xu et al. identified competence, intrinsic regulation and identified regulation as the SDT constructs most consistently associated with PA across age groups, recommending that competence and autonomous motivation be prioritised over external pressures or rewards [18].

In the present study, SDT is used primarily as a framework for understanding motivational regulation rather than as a direct test of basic psychological need satisfaction. This distinction is important because evidence on the motivational processes related to PA is limited among pwCF. A recently published study found that intrinsic motivation was the strongest predictor of enjoyment of PA in adults with CF [21]. More recently, Welsner et al. reported that PA enjoyment fully mediates the relationship between ppFEV₁ and vigorous PA in adult pwCF, highlighting the central role of affective factors in PA behaviour [22].

Nevertheless, the relationship between pulmonary function, the spectrum of motivational regulation, and PA enjoyment in adult pwCF remains unclear, particularly when relevant clinical factors such as CFTR modulator therapy status or sex are considered [8, 9, 23, 24].

The present study investigated the associations between pulmonary function (ppFEV_1_), motivational regulation, and the enjoyment of PA in adult pwCF. Based on SDT and previous PA research in CF, we hypothesised that the ppFEV₁ would be positively and independently associated with more autonomous forms of motivational regulation and greater enjoyment of PA, regardless of CFTR modulator therapy status and sex. Because of the cross-sectional design, this hypothesis was examined for its association rather than its causal or mechanistic pathway.

### Materials and methods

A cross-sectional study design was used and data were collected at the Adult Cystic Fibrosis Centre of the Ruhrlandklinik, Essen, Germany. Eligible participants were adults aged 18 years or older who had received a confirmed clinical diagnosis of CF according to standard diagnostic criteria. This diagnosis was based on positive sweat chloride test results, characteristic clinical manifestations and, where available, documentation of pathogenic CFTR variants. Where documented, the CFTR genotype was recorded as homozygous F508del, heterozygous F508del or other combinations of pathogenic variants. In five participants (2.5%), specific genotype information was unavailable in the medical records at the time of data collection, despite the clinical CF diagnosis being firmly established by a positive sweat chloride test.

The present analysis is based on the recruitment cohort of adults with CF at the Adult Cystic Fibrosis Centre of the Ruhrlandklinik, Essen, which was also the basis for two previous publications from our group that addressed different research questions [21, 22]. There is a high level of overlap in participants: all 197 participants from the Gruber et al. study and 167 of the 168 participants from the Welsner et al. study are included in the present sample of 200 adults, with 167 participants shared across all three analyses.

However, the three studies differ fundamentally in terms of their research questions and dependent variables. Gruber et al. modelled enjoyment of PA as the outcome variable, while Welsner et al. considered PA behaviour as the outcome variable with enjoyment of PA acting as a mediator [21, 22]. The present analysis models the BREQ-2 motivational regulation subscales and the RAI as the outcomes, with ppFEV₁ serving as an independent clinical correlate. Neither previous publication by the working group examined the specific research question of whether pulmonary function is independently associated with autonomous motivational regulation. A detailed comparison of samples, variables and analyses can be found in Supplementary Table S1.

All participants were invited to participate during their scheduled outpatient appointments.

After providing written informed consent, participants completed a standardised set of validated self-report questionnaires covering the following two aspects: (1) PA and (2) the motivational regulation of PA. The two questionnaires used in this study, the PACES and the BREQ-2, are previously published and validated instruments that were not developed for this study [25–27]. For the present analyses, only participants with complete data for the variables required in the current models were included in the final analytical sample.

The relevant clinical parameters were extracted from the medical records of each participant. These included ppFEV₁, percent predicted forced vital capacity (ppFVC), age (years), height (cm), body weight (kg), body mass index (BMI, kg/m^2^), pancreatic status (sufficient or insufficient), current CFTR modulator therapy, the presence of cystic fibrosis-related diabetes (CFRD), transplantation history (lung (LuTx) or liver (LTx)), and CFTR genotype.

For pragmatic and conceptual reasons, the ppFEV₁ was selected as the primary clinical variable of interest. It is a widely standardised and routinely available marker of pulmonary disease severity in CF that is commonly recorded during routine clinical care and included in national and international CF registries [28–30]. Therefore, ppFEV₁ is a clinically interpretable parameter frequently used to contextualise disease status and inform clinical decisions. In contrast, more specialised, physiologically proximal measures of exercise capacity, such as VO₂peak, other cardiopulmonary exercise testing (CPET) parameters or the six-minute walk test (6MWT), were unavailable in the present dataset. Consequently, ppFEV₁ was examined as a routinely available clinical correlate of motivational regulation, rather than as a presumed causal or mechanistically specific determinant.

In this study, the term „PA “ is used as an umbrella term. Consistent with Piggin's holistic definition, PA encompasses not only bodily movement and energy expenditure, but also how people move, act and perform in culturally specific spaces and contexts. This definition incorporates the influence of interests, emotions, ideas, instructions and relationships [31]. According to Caspersen et al., exercise is a specific subset of PA, and refers to planned, structured, and repetitive activities aimed at improving or maintaining physical fitness [32].

Throughout the manuscript, 'PA' is used as the broader term, whereas 'exercise' is reserved for planned, structured activity and exercise-specific wording in the BREQ-2. It is also used in contexts where cited studies use exercise-specific terminology. The BREQ-2 and PACES were administered without modification. PACES outcomes are described as PA enjoyment, consistent with the wording of the German PACES items used in this study.

### Questionnaires

#### Enjoyment

Enjoyment of PA was assessed using the 16-Item German version of the Physical Activity Enjoyment Scale (PACES) [25]. The questionnaire is based on the revision by Motl et al. and uses a five-point Likert scale ranging from 1 (strongly disagree) to 5 (strongly agree). Sample items include 'When I am physically active, I feel joy' and 'When I am physically active, I enjoy it’ [33].

The German adult version has shown high internal consistency (Cronbach's α = 0.94) [25]. Total scores were calculated by summing item responses, which ranged from 16 to 80 points. Higher scores reflect greater enjoyment of PA. As there is no universally accepted clinical cut-off point for adults with CF, the terms “low”, “moderate” and “high” are used descriptively rather than diagnostically.

#### Motivation

The German version of the Behavioural Regulation in Exercise Questionnaire-2 (BREQ-2) was used to evaluate motivation for PA. This questionnaire has shown acceptable internal consistency across its subscales (Cronbach's α = 0.62–0.90) [27]. The instrument comprises 19 items that are rated on a five-point Likert scale (1 = not at all true for me; 5 = very true for me) [27].

The BREQ-2 assesses five distinct types of motivational regulation that reflect gradually increasing levels of self-determination. Amotivation (four items; e.g., "I do not see why I should have to exercise") represents a complete lack of motivation, where the individual sees no reason to engage in PA. External regulation (four items; e.g., "I exercise because other people say I should") reflects behaviour driven entirely by external pressure, rewards or demands from others. Introjected regulation (three items; e.g., "I feel guilty when I do not exercise") is internalised but remains controlled, being driven by self-imposed pressure such as guilt or shame rather than by external factors.

The identified regulation (four items, e.g. „I value the benefits of exercise “) reflects a greater sense of autonomy, where the individual consciously recognises and endorses the personal importance of PA. Intrinsic motivation (four items; e.g. „I find exercise a pleasurable activity “) is the most self-determined type of motivational regulation and is characterised by the inherent enjoyment and satisfaction derived from the activity itself [34].

In the current BREQ-2-based analyses, autonomous motivation is represented by identified regulation and intrinsic motivation. This refers to the extent to which PA and exercise is experienced as a freely chosen, personally endorsed and self-determined activity, rather than being driven by external or internal pressure [35].

The subscale score for each regulation type was computed by averaging the corresponding item responses, with higher scores indicating stronger adherence to that regulation type. Additionally, a composite score, the relative autonomy index (RAI), was derived using the following formula: RAI = (3 * intrinsic motivation) + (2 * identified regulation) + (− 1 * introjected regulation) + (− 2 * external regulation) + (− 3 * amotivation).

Because the RAI is a relative continuum score, it does not provide a validated clinical threshold above which a person can be classified as having an autonomous motivational profile. In this study, RAI values are therefore interpreted descriptively and comparatively, rather than categorically. Higher RAI values indicate a greater degree of autonomous motivation overall [27].

#### Statistics

All data are presented as the mean ± standard deviation (SD). Where appropriate, 95% confidence intervals (CIs) are reported for model-based estimates. Prior to statistical analysis, the distribution of all study variables was assessed using the Shapiro–Wilk test and visual inspection of Q–Q plots. With respect to the regression-based analyses, the assumptions of the models were further evaluated by inspecting the residuals for normality (Q-Q plots of residuals), linearity and homoscedasticity (residual versus predicted plots). No rank-based analyses were performed in the final analyses.

A multivariate general linear model (GLM) was used to analyse differences between groups in terms of anthropometric parameters, motivational regulation and enjoyment of PA. CFTR modulator treatment and sex were included as fixed between-subject factors and ppFEV₁ was used as a continuous covariate to examine its association with motivational and enjoyment outcomes, adjusting for group-level factors. CFTR modulator therapy status and sex were used a priori as adjustment variables because they were the group-level factors most directly relevant to the research question. Owing to the sample size and number of outcomes, the models were kept as simple as possible to avoid overfitting. Therefore, additional clinical variables were not included as covariates in the primary models.

As an exploratory sensitivity analysis, the multivariate GLM was repeated without ppFEV₁ being considered as a covariate, in order to examine whether adjusting for lung function would affect the pattern of sex-related effects. An additional exploratory sensitivity analysis involved repeating the hierarchical regression models for intrinsic motivation, the RAI and the PACES total score, entering age and BMI as additional covariates, to determine whether the associations between ppFEV₁ and the main outcomes remained significant after adjustment for these variables. Effect sizes are reported as partial eta-squared (η^2^p).

To examine the associations between ppFEV₁ and motivational and enjoyment outcomes, independently of CFTR modulator therapy status and sex, partial correlations were calculated between ppFEV₁ and BREQ-2 subscale scores, as well as the RAI and PACES total scores, while controlling for CFTR modulator therapy status and sex. Although several variables deviated from normality, parametric analyses (general linear modelling, partial correlations and hierarchical regression) were used because the sample size was large enough (*N* = 200) and model assumptions were evaluated using residual diagnostics.

Hierarchical multiple regression analyses were performed with intrinsic motivation, the RAI and the PACES total scores serving as the dependent variables. In the first block, CFTR modulator treatment and sex were entered as control variables. In the second block, ppFEV₁ was entered as the clinical variable of interest. The change in explained variance (ΔR^2^) was used to quantify the additional variance associated with ppFEV₁ in addition to that accounted for by the control variables. Multicollinearity among the variables in the primary models was assessed using variance inflation factors (VIFs) and tolerance statistics.

To address potential residual confounding factors, extended exploratory sensitivity analyses were conducted for the three main outcomes, intrinsic motivation, the RAI and PA enjoyment. In these models, age, BMI and available clinical disease-burden markers were added to the original set of adjustments. The additional covariates included CFRD, Pseudomonas aeruginosa colonisation, pancreatic status, and genotype category. Genotype category was coded as F508del homozygous versus non-F508del homozygous, and participants for whom genotype information was unavailable were treated as missing data. Therefore, the extended sensitivity analyses including genotype category were based on 195 participants. Transplantation history was not included in the extended models due to sparse cell counts and the risk of unstable estimates. The ppFEV₁ was included in the final block to determine whether it explained additional variance following broader clinical adjustment. Multicollinearity in the extended sensitivity models was assessed using variance inflation factors (VIFs) and tolerance statistics.

Statistical significance was set to p < 0.05 (two-tailed). All analyses were performed using SPSS version 30.0 (IBM Corp., Armonk, NY, USA).

## Results

**Fig. 1:**
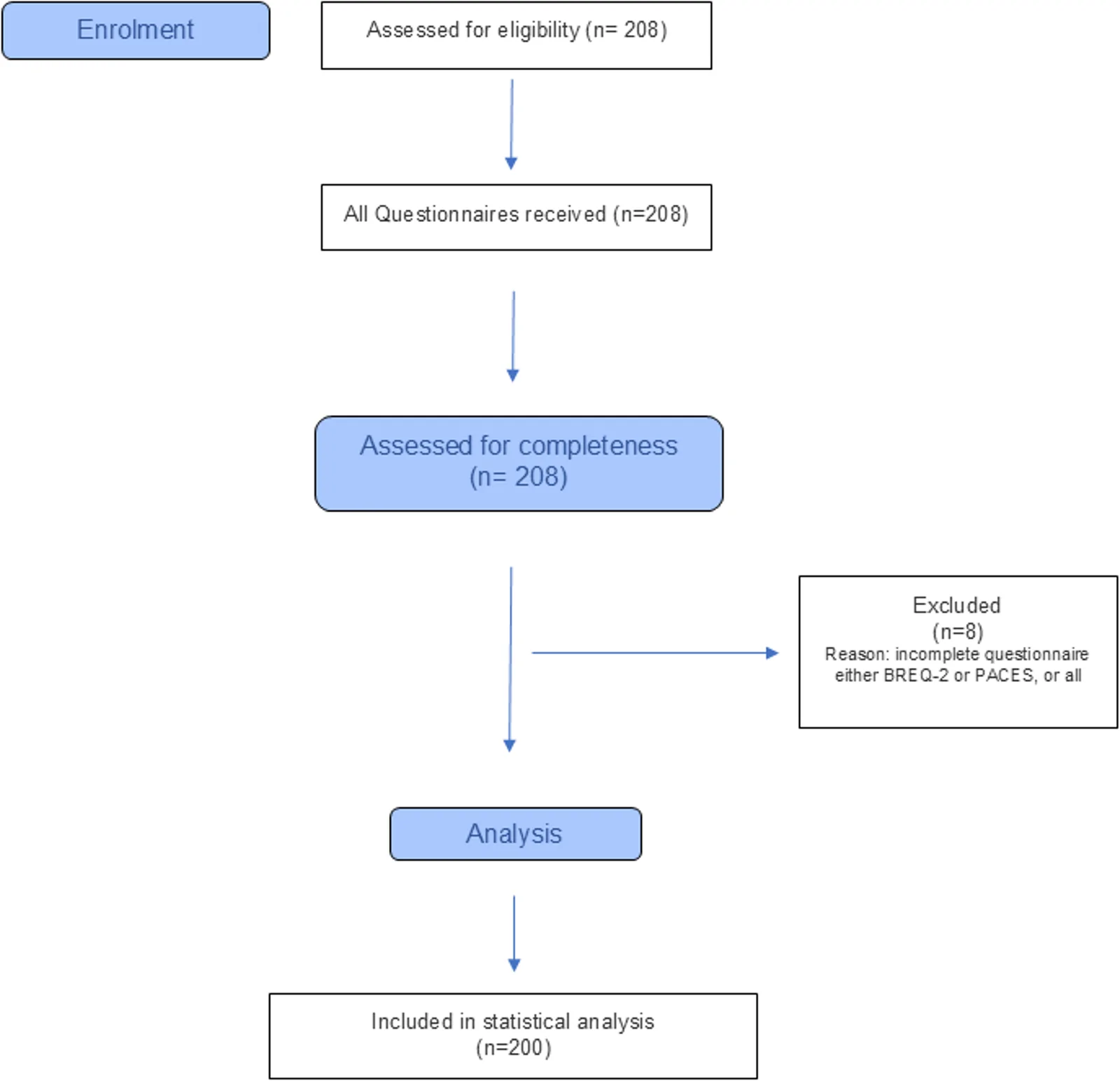
Participant flow diagram. Of the 208 adults with CF who were assessed for eligibility, all returned questionnaires. Eight were excluded because of incomplete or invalid BREQ-2 or PACES responses, leaving 200 participants who were included in the statistical analysis. The CFTR genotype category was unavailable for five of these participants (2.5%), so the exploratory sensitivity analyses including this category (see Supplementary Table S2) were based on *n* = 195

All 208 adult pwCF assessed for eligibility at the Adult Cystic Fibrosis Centre of the Ruhrlandklinik, Essen, returned completed questionnaire sets. Eight participants were subsequently excluded because of incomplete or invalid responses on either the BREQ-2 or the PACES questionnaire, resulting in a final analytical sample of *n* = 200 (Fig. 1).

### Sample characteristics

A total of 200 adult pwCF were included in the analysis. Their demographic and clinical characteristics are summarised in Table 1. The sample comprised 117 males (58.5%) and 83 females (41.5%) with a mean age of 35.8 ± 12.3 years. The mean ppFEV₁ was 71.2 ± 22.4%, indicating mild-to-moderate pulmonary impairment on average. Most participants (*n* = 175, 87.5%) received CFTR modulator therapy (including mono-, dual-, or triple therapy). The most common CFTR genotype was homozygous F508del (*n* = 89, 44.5%), followed by heterozygous F508del (*n* = 82, 41.0%). Other mutations accounted for 24 participants (12.0%), and genotype information was unavailable for 5 participants (2.5%). Pancreatic insufficiency affected 86.8% of the cohort, whereas Pseudomonas aeruginosa colonisation was documented in 49.8% of the cohort.

**Table 1:** Demographic data and clinical outcomes

	pwCF (n = 200)	
**Characteristics**		95.0% CI (LL/UL)
**Demographics**		
male, n (%)	117 (58.5)	
female, n (%)	83 (41.5)	
age (years), mean (SD)	35.8 (12.3)	34.1/37.5
heigth (cm), mean (SD)	171.7 (9.2)	170.4/173.0
weight (kg), mean (SD)	70.0 (15.4)	67.9/72.1
BMI (kg/m^2^), mean (SD)	23.8 (4.5)	23.1/24.1
**Pulmonary Function**		
ppFEV₁, mean (SD)	71.2 (22.4)	68.1/74.3
ppFVC, mean (SD)	87.3 (18.8)	84.8/89.9
**Genetics**		
del F508 homozygous, n (%)	89 (44.5)	
del F508 heterozygous, n (%)	82 (41.0)	
other mutations, n (%)	24 (12.0)	
no information n (%)	5 (2.5)	
**Treatment**		
CFTR-Modulator (mono, dual or triple combination), n (%)	175 (87.5)	
**Comorbidities**		
Pancreatic insufficiency, n (%)	178 (89.0)	
CFRD, n (%)	17 (8.5)	
Pseudomonas aeruginosa, n (%)	102 (51.0)	
**Transplantation**		
LuTx, n (%)	1 (0.5)	
LTx, n (%)	8 (4.0)	

Data are presented as mean (SD) and 95% CI for continuous variables, and as n (%) for categorical variables. 95% CI = confidence interval (lower to upper limit); CFRD = Cystic Fibrosis Related Diabetes, LuTx = lung transplantation, LTx = liver transplantation; *BMI* Body Mass Index, *ppFEV*₁ Percent predicted forced expiratory volume in 1 s, *ppFVC* Percent predicted forced vital capacity

### Motivational regulation and PA enjoyment

The descriptive statistics for the BREQ-2 subscales, the RAI and the PACES total score are listed in Table 2. Identified regulation (M = 3.4 ± 1.0) and intrinsic motivation (M = 3.7 ± 1.2) yielded the highest mean scores, whereas amotivation (M = 1.3 ± 0.7) and external regulation (M = 1.4 ± 0.6) yielded the lowest scores. The mean RAI score was 9.0 ± 7.1 and the mean PACES-assessed PA enjoyment score was 65.5 ± 11.2 (possible range 16–80).

**Table 2:** BREQ-2 and PACES Questionnaire Results in pwCF

	pwCF (n = 200)	
	mean (SD)	95.0% CI (LL/UL)
**BREQ-2 Subscales**		
Mean Amotivation	1.3 (0.7)	1.2/1.4
Mean External Regulation	1.4 (0.6)	1.3/1.5
Mean Introjected Regulation	2.1 (0.9)	2.0/2.3
Mean Identified Regulation	3.4 (1.0)	3.3/3.6
Mean Intrinsic Regulation	3.7 (1.2)	3.5/3.8
Relative Autonomy Index (RAI)	9.0 (7.1)	8.0/9.9
**PACES**		
Enjoyment (total score)	65.5 (11.2)	64.0/67.0

Data are presented as mean (SD); 95% CI = confidence interval (lower to upper limit); BREQ-2 = Behavioural Regulation in Exercise Questionnaire-2; PACES = Physical Activity Enjoyment Scale; BREQ-2 scoring: Each subscale score calculated as mean of respective items (range 1–5), with higher scores indicating stronger regulation type; The RAI represents the degree of self-determined motivation, with higher positive values indicating more autonomous motivation and negative values indicating more controlled motivation; PACES scoring: Sum of all 16 items, with higher scores indicating greater physical activity enjoyment

### Multivariate General Linear Model (GLM)

The results of the multivariate GLM are listed in Table 3. When CFTR modulator therapy status and sex were included as fixed between-subject factors and ppFEV₁ as a continuous covariate, neither CFTR modulator therapy status nor sex had a significant effect on any of the motivational or enjoyment outcomes (all *p* > 0.05). No significant interaction was observed between sex and CFTR modulator therapy for any dependent variable (all *p* > 0.05).

**Table 3:** Multivariate GLM – Tests of Between-Subjects Effects (*N* = 200)

Source	Dependent variable	F	p	η^2^p
ppFEV₁	Mean Amotivation	3.353	.069	.017
	Mean External Regulation	0.702	.403	.004
	Mean Introjected Regulation	1.248	.265	.006
	Mean Identified Regulation	**5.107**	**.025**	**.026**
	Mean Intrinsic Regulation	**10.899**	**.001**	**.053**
	Relative Autonomy Index	**8.068**	**.005**	**.040**
	PACES total score	**8.058**	**.005**	**.040**
Sex	Mean Amotivation	0.172	.679	.001
	Mean External Regulation	0.000	.994	.000
	Mean Introjected Regulation	1.100	.296	.006
	Mean Identified Regulation	0.551	.459	.003
	Mean Intrinsic Regulation	0.676	.412	.003
	Relative Autonomy Index	0.041	.840	.000
	PACES total score	0.001	.972	.000
CFTR modulator therapy	Mean Amotivation	0.404	.668	.004
	Mean External Regulation	0.309	.734	.003
	Mean Introjected Regulation	1.350	.262	.014
	Mean Identified Regulation	0.892	.412	.009
	Mean Intrinsic Regulation	0.238	.789	.002
	Relative Autonomy Index	0.556	.575	.006
	PACES total score	0.945	.390	.010

Bold values indicate statistical significance (*p* <.05). η^2^p = partial eta-squared; PACES = Physical Activity Enjoyment Scale; RAI = Relative Autonomy Index; CFTR modulator therapy: three groups (ETI, mono/dual modulator, no modulator); df = 1 for ppFEV₁ and sex; df = 2 for CFTR modulator therapy

In contrast, ppFEV₁ was significantly associated with several motivational outcomes. Specifically, ppFEV₁ was significantly associated with intrinsic motivation (F(1, 193) = 10.899, *p* = 0.001, η^2^p = 0.053), the RAI (F(1, 193) = 8.068, *p* = 0.005, η^2^p = 0.040), the PACES total score (F(1, 193) = 8.058, *p* = 0.005, η^2^p = 0.040) and identified regulation (F(1, 193) = 5.107, *p* = 0.025, η^2^p = 0.026). The association with amotivation did not reach statistical significance (F(1, 193) = 3.353, *p* = 0.069, η^2^p = 0.017). No significant associations were observed between ppFEV₁ and external or introjected regulation (both *p* > 0.26).

An additional exploratory sensitivity GLM was performed, excluding ppFEV₁ as a covariate. However, it is not presented in the tables. Sex remained non-significant across all BREQ-2 subscales, the relative autonomy index, and the PACES total score. All p-values were ≥ 0.248, and the effect sizes were small or negligible, with partial η^2^ ≤ 0.007.

### Partial correlations

Partial correlations were calculated while controlling for CFTR modulator therapy status and sex (see Table 4). ppFEV₁ was positively associated with intrinsic motivation (*r* = 0.236, *p* < 0.001), the RAI (*r* = 0.204, *p* = 0.004), the PACES total score (*r* = 0.202, *p* = 0.004) and identified regulation (*r* = 0.159, *p* = 0.025). The negative association with amotivation did not reach statistical significance (*r* = −0.134, *p* = 0.059).

**Table 4:** Partial Correlations between ppFEV₁ and Motivational Regulation and PA Enjoyment, Controlling for CFTR Modulator Therapy Status and Sex (*N* = 200)

Variable	Amotivation	External Regulation	Introjected Regulation	Identified Regulation	Intrinsic Regulation	RAI	PACES total score
ppFEV₁ (% predicted)							
r	−1.37	-.060	.073	.159*	.236**	.204**	.202**
p	(.059)	(.397)	(.309)	(.025)	(<.001)	(.004)	(.004)
Mean Amotivation							
r	—	.179*	-.265**	-.562**	-.597**	-.752**	-.541**
p		(.012)	(<.001)	(<.001)	(<.001)	(<.001)	(<.001)
Mean External Regulation							
r	—	—	.027	-.312**	-.387**	-.516**	-.413**
p			(.708)	(<.001)	(<.001)	(<.001)	(<.001)
Mean Introjected Regulation							
r	—	—	—	.435**	.220**	.179*	.171*
p				(<.001)	(.002)	(.012)	(.016)
Mean Identified Regulation							
r	—	—	—	—	.783**	.843**	.627**
p					(<.001)	(<.001)	(<.001)
Mean Intrinsic Regulation							
r	—	—	—	—	—	.939**	.791**
p						(<.001)	(<.001)
Relative Autonomy Index (RAI)							
r	—	—	—	—	—	—	.785**
p							(<.001)
PACES total score							

^*^p <.05; ** *p* <.001. *r* = partial correlation coefficient, controlling for CFTR modulator therapy status and sex; *RAI* Relative Autonomy Index, *PACES *Physical Activity Enjoyment Scale. Partial correlations were computed with df = 196

### Hierarchical regression analyses

Three hierarchical regression analyses were conducted to quantify the additional variance in intrinsic motivation, RAI and PACES total scores associated with ppFEV₁, independent of CFTR modulator therapy status and sex.

The results are summarised in Table 5.

**Table 5:** Hierarchical Regression Analyses – ppFEV₁ as Correlate of Autonomous Motivation and PA Enjoyment (*N* = 200)

Dependent variable	Block 1: CFTR + sex		Block 2: + ppFEV₁			ppFEV₁ coefficient			
	R^2^	p	R^2^	ΔR^2^	p (ΔR^2^)	B	95% CI (LL/UL)	β	p
Mean Intrinsic Regulation	.022	.117	.076	.054	** <.001**	.012	.005/.019	.233	** <.001**
Relative Autonomy Index (RAI)	.013	.277	.054	.041	**.004**	.064	.021/.108	.203	**.004**
PACES total score	.016	.201	.056	.040	**.004**	.101	.032/.169	.201	**.004**

Bold values indicate statistical significance (*p* <.05). R^2^ Coefficient of determination, ΔR^2^ Change in R^2^ when ppFEV₁ entered in Block 2, β Standardised regression coefficient, *CFTR*_CFTR modulator therapy (three groups: ETI, mono/dual, no modulator), *PACES* Physical Activity Enjoyment Scale. *RAI* Relative Autonomy Index. B unstandardised regression coefficient, *95% CI* 95% confidence interval for B

In all three models, Block 1 (CFTR modulator therapy status and sex) was not a significant factor in explaining the variance of any of the three outcomes (intrinsic motivation: R^2^ = 0.022, *p* = 0.117; RAI: R^2^ = 0.013, *p* = 0.277; and PACES: R^2^ = 0.016, *p* = 0.201). Block 2 significantly increased the explained variance for intrinsic motivation (ΔR^2^ = 0.054, *p* < 0.001; β = 0.233, *p* < 0.001), the RAI (ΔR^2^ = 0.041, *p* = 0.004; β = 0.203, *p* = 0.004), and the PACES (ΔR^2^ = 0.040, *p* = 0.004; β = 0.201, *p* = 0.004). However, CFTR modulator therapy status and sex remained non-significant in all three models. Additional exploratory sensitivity analyses were conducted to examine whether the associations between ppFEV₁ and the three main outcomes remained consistent when further adjustments were made for age, BMI, and available clinical disease burden markers. In these models, ppFEV₁ remained significantly associated with intrinsic motivation (β = 0.240, B = 0.013, 95% CI 0.005 to 0.021, *p* = 0.002), the RAI (β = 0.197, B = 0.063, 95% CI 0.014 to 0.112, *p* = 0.012) and PA enjoyment (β = 0.232, B = 0.118, 95% CI 0.040 to 0.195, *p* = 0.003). The additional variance explained by ppFEV₁ was small but statistically significant across the outcomes: 4.8% for intrinsic motivation, 3.2% for the RAI, and 4.5% for PA enjoyment.

No relevant multicollinearity was observed in either the primary or the extended sensitivity regression models, with all VIF values below 1.01 in the former and a maximum of 1.349 in the latter. The full results of the extended sensitivity analyses are presented in Supplementary Table S2.

## Discussion

This cross-sectional study examined the relationship between pulmonary function (ppFEV₁), motivational regulation, and PA enjoyment in 200 adult pwCF. After adjusting for CFTR modulator therapy status and sex, ppFEV₁ was significantly associated with autonomous motivational regulation and PA enjoyment. The effect sizes were modest (ΔR^2^ = 0.040–0.054), indicating that ppFEV₁ only explained a small additional proportion of the variance in these outcomes, suggesting that other psychological, social, environmental, or clinical factors may also contribute. Due to the cross-sectional design, these findings describe associations and do not establish temporal ordering or causal mechanisms.

The descriptive findings revealed that identified and intrinsic regulation had the highest mean scores, while amotivation and external regulation had the lowest. For comparison, mean RAI values of between 10 and 11, together with similarly high intrinsic and identified regulation scores, have previously been reported in adults with rheumatoid arthritis [36]. However, direct comparisons across chronic disease populations are limited by differences in study design and sample characteristics. Nevertheless, the present CF sample shows a similar pattern of relatively greater autonomous regulation. The RAI and PACES values should be interpreted descriptively rather than as clinical categories, as validated cut-offs for adults with CF are not yet available. It remains to be tested whether SDT-informed interventions can further support autonomous motivation and PA enjoyment.

According to SDT, it is theoretically plausible that a better-preserved ppFEV₁ is associated with experiences that are relevant to perceived competence during PA. Satisfying the need for competence, feeling effective and capable during PA, is important for internalising motivation and developing autonomous regulation [17]. Individuals with better-preserved pulmonary function may experience fewer ventilatory constraints, and perceive themselves as more physically capable, thereby deriving greater intrinsic satisfaction and enjoyment. This interpretation is consistent with SDT-informed PA research, in which competence satisfaction is associated with autonomous motivation and PA enjoyment [18–20]. As perceived competence was not assessed, and no mediation model was tested, this remains a theoretical account.

It should be noted that ppFEV₁ reflects the degree of airway obstruction and should not be interpreted as a direct indicator of exercise capacity, perceived physical competence, ventilatory limitations or symptom burden during PA [2, 9]. Proximal physiological, functional, and psychological measures, such as VO₂peak and other CPET parameters, the 6MWT, perceived competence, and dyspnoea during activity, were unavailable in the present dataset and could therefore not be examined as alternative explanatory variables or potential mediators. The observed associations may therefore reflect pulmonary function specifically, but they may also partly capture broader disease severity or clinical burden.

Against this background, the present findings can be viewed in relation to earlier work from the same centre. Gruber et al. reported that intrinsic motivation was the strongest predictor of enjoyment of PA, as measured by the PACES [21]. Additionally, Welsner et al. demonstrated that enjoyment of PA, as also assessed by the PACES, mediates the relationship between ppFEV₁ and self-reported PA levels [22]. The present analysis complements these findings by addressing a different question. Rather than modelling predictors of enjoyment or the behavioural consequences of enjoyment, this study examines whether pulmonary function is associated with motivational regulation itself.

Specifically, this is the first analysis in this cohort to examine the individual BREQ-2 motivational regulation subscales, particularly intrinsic motivation and the RAI as outcomes in their own right. To our knowledge, no previous studies in the CF literature have reported associations between ppFEV₁ and autonomous motivational regulation. Therefore, the present findings extend previous work from the same centre by linking pulmonary function not only to PA enjoyment and PA behaviour, but also to the broader motivational regulation profile underlying PA engagement.

Neither CFTR modulator therapy status nor its interaction with sex showed a statistically significant association with any motivational or enjoyment outcome. This suggests that CFTR modulator therapy status alone is not sufficient to capture individual differences in motivational regulation or PA enjoyment. From an SDT perspective, improvements in exercise capacity alone would not be expected to ensure the satisfaction of psychological needs such as autonomy, competence and relatedness in the context of PA [17].

These findings have practical implications for motivational support in the era of highly effective CFTR modulator therapy. Although CFTR modulators, particularly ETI, have produced substantial improvements in pulmonary function and other clinical outcomes [1, 5–7], habitual PA does not appear to change consistently in the modulator era [10], and sustained engagement in PA has been identified as a continuing challenge in contemporary CF care [9]. Motivational and psychological factors may therefore remain relevant to sustained PA participation, even as some of the physiological limitations associated with CF become less prominent.

In the present GLM, no statistically significant main effect of sex was observed for any motivational or enjoyment outcome. These findings are consistent with those of a meta-analysis by Guérin et al., who reported negligible gender differences at the mean level across BREQ/BREQ-2 motivational regulations and the RAI [37]. Direct comparability should be interpreted with caution, because this meta-analysis primarily examined mean-level differences in non-clinical PA samples, rather than covariate-adjusted associations in clinical populations [37]. At the individual-study level, the data are mixed. For example, Duncan et al. reported that integrated and identified regulation were associated with exercise frequency in both men and women, whereas introjected regulation was only associated with exercise intensity in women [38]. With regard to the enjoyment of PA, Jekauc et al. reported measurement invariance across sex in their work on the German adult version of the PACES, thereby supporting the idea of valid comparisons between men and women [25].

Exploratory sensitivity analyses that excluded ppFEV₁ as a covariate also showed no main effect of sex. This indicates that the absence of a significant finding was not solely due to the adjustment for lung function. However, this does not imply that sex is irrelevant to motivational processes in CF. Further research with adequate statistical power is needed to establish whether sex influences the relationship between lung function and motivational outcomes.

Clinically, and in light of the cross-sectional design and modest effect sizes, ppFEV₁ should not be used in isolation to identify pwCF in need of motivational support. However, it may provide useful contextual information when discussing PA participation. Motivational regulation and PA enjoyment should be assessed directly at the individual level. This could inform a decision as to whether support should focus on strengthening autonomous motivation, reducing controlled forms of regulation, increasing enjoyment, or adapting PA opportunities to the individual's functional capacity and daily context. Accordingly, SDT-informed counselling and PA interventions could be designed to promote competence and autonomous motivation, and to make PA meaningful, achievable and enjoyable for people with CF [18–20].

The study has several limitations. Firstly, the cross-sectional design precludes causal inference and temporal ordering. The observed associations between ppFEV₁ and motivational and enjoyment outcomes could be explained in several ways, including the effect of pulmonary status on motivational experiences, reverse or bidirectional relationships, or the influence of unmeasured clinical, behavioural or psychosocial factors. While the extended exploratory sensitivity analyses, which adjusted for age, BMI, and available clinical disease burden markers, showed that the associations between ppFEV₁ and the three main outcomes remained statistically significant, residual confounding by unmeasured factors, such as symptom burden, psychosocial resources, and habitual PA, cannot be excluded. Therefore, longitudinal studies are needed to establish temporal ordering and investigate potential mechanisms.

Secondly, the group of participants not receiving CFTR modulator therapy was small (*n* = 25), which reduced the statistical power to detect potential group differences in motivational and enjoyment outcomes. Accordingly, the absence of a statistically significant CFTR modulator therapy effect should be interpreted with caution and examined in larger, more balanced groups.

Thirdly, the dataset did not include a validated measure of basic psychological need satisfaction. This prevented direct assessment of the proposed competence-based interpretation, as well as of basic need satisfaction more broadly. Future studies should incorporate validated measures of autonomy, competence, and relatedness in PA or exercise contexts, such as the Basic Psychological Needs in Exercise Scale [39]. Furthermore, the BREQ-2 does not include an integrated regulation subscale, resulting in an incomplete representation of the full motivational continuum conceptualised within SDT [34].

Fourthly, the present analysis did not include an objective or sufficiently validated measure of actual PA behaviour. Consequently, it cannot be determined whether greater autonomous motivation or PA enjoyment translated into higher levels of habitual PA, structured exercise participation or adherence to PA recommendations. This is particularly relevant because SDT-based research has consistently linked more autonomous forms of motivation to exercise and PA behaviour [19]. Future studies should therefore combine motivational and affective measures such as the BREQ-2 and PACES with objective PA monitoring or validated behavioural PA measures.

Fifthly, the motivational and enjoyment outcomes in this cohort were not evaluated in terms of measurement error, test–retest reliability and the minimal detectable change. Therefore, it remains unclear whether the observed associations exceed measurement error at the individual level, or whether they are significant enough to inform clinical decisions.

Finally, the present study and the complementary studies by Gruber et al. and Welsner et al. were conducted at the same CF centre and are based on substantially overlapping samples [21, 22]. These findings therefore cannot be considered an independent replication across separate cohorts. The single-centre design may also limit the generalisability of the findings. Confirmation in independent, prospective, multicentre cohorts is warranted.

## Conclusion

In adult pwCF, ppFEV₁ was independently associated with autonomous motivational regulation and PA enjoyment, after adjusting for CFTR modulator therapy status and sex. However, as these associations were derived from cross-sectional, single-centre data, no causal or directional conclusions can be drawn, and their magnitude is unlikely to be sufficient to justify using ppFEV₁ as a proxy for motivational support needs or as a basis for personalised clinical decision-making. Motivational regulation and PA enjoyment should therefore be assessed directly across the full spectrum of pulmonary function.

Furthermore, CFTR modulator therapy status was not associated with motivational or enjoyment outcomes, suggesting that treatment status alone does not capture individual differences in these psychological outcomes. As the clinical status of many pwCF improves, directly assessing motivational regulation and PA enjoyment may remain important in routine care. Such assessments could inform person-centred, SDT-informed support aimed at strengthening autonomous motivation and enjoyment as components of comprehensive CF care.

## Supplementary Information

Supplementary Material 1.

Supplementary Material 2.

## Data Availability

The data presented in this study are subject to the terms of data protection laws. Thus, these data are only available upon reasonable request and in justified cases, from the corresponding authors.

## Abbreviations

6MWT: Six-Minute Walk Test
BMI: Body Mass Index
BREQ-2: Behavioural Regulation in Exercise Questionnaire-2
CF: Cystic Fibrosis
CFRD: Cystic Fibrosis-Related Diabetes
CFTR: Cystic Fibrosis Transmembrane Conductance Regulator
CI: Confidence Interval
CPET: Cardiopulmonary Exercise Testing
ETI: Elexacaftor/Tezacaftor/Ivacaftor
GLM: General Linear Model
LTx: Liver Transplantation
LuTx: Lung Transplantation
MVPA: Moderate-to-Vigorous Physical Activity
PA: Physical Activity
PACES: Physical Activity Enjoyment Scale
ppFEV₁: Percent Predicted Forced Expiratory Volume in One Second
ppFVC: Percent Predicted Forced Vital Capacity
pwCF: People with Cystic Fibrosis
RAI: Relative Autonomy Index
SD: Standard Deviation
SDT: Self-Determination Theory
VIF: Variance Inflation Factor
VO₂peak: Peak Oxygen Uptake
