# Supplementary material for: Moving beyond treatment: pulmonary function, autonomous motivation, and physical activity enjoyment in adults with cystic fibrosis
Source: BMC Pulm Med. 2026 Sep 7;26:400. doi: 10.1186/s12890-026-04661-w (PMC13548599; doi:10.1186/s12890-026-04661-w)
Supplement: Supplementary file 1 [file 12890_2026_4661_MOESM1_ESM.docx]

**Supplementary Table S1. Comparison of the present manuscript with previous publications from the same centre**

| **Aspect** | **Gruber et al., Healthcare 2025 [1]** | **Welsner et al., Frontiers in Sports and Active Living 2026 [2]** | **Present manuscript** |
| --- | --- | --- | --- |
| Publication / title | Predictors of Physical Activity Enjoyment in Adults with Cystic Fibrosis: The Role of Quality of Life and Motivation - A Single-Center Study. | Mediating effects of physical activity enjoyment on physical activity levels in adults with cystic fibrosis. | Moving Beyond Treatment: Pulmonary Function, Autonomous Motivation, and Physical Activity Enjoyment in Adults with Cystic Fibrosis. |
| Sample source | Adults with Cystic Fibrosis (CF) recruited Department of Pulmonary Medicine, University Hospital Essen - Ruhrlandklinik, Adult Cystic Fibrosis Center, University of Duisburg-Essen, Essen, Germany | Adults with CF recruited Department of Pulmonary Medicine, University Hospital Essen - Ruhrlandklinik, Adult Cystic Fibrosis Center, University of Duisburg-Essen, Essen, Germany | Adults with CF recruited Department of Pulmonary Medicine, University Hospital Essen - Ruhrlandklinik, Adult Cystic Fibrosis Center, University of Duisburg-Essen, Essen, Germany |
| Analytical sample size | n = 197 adult pwCF. | n = 168 adult pwCF with complete PACES and 7-day Physical Activity Recall data. | n = 200 adult pwCF with complete BREQ-2 and PACES data required for the current models. |
| Participant overlap with the present manuscript | There are overlaps with the present manuscript. Both studies drew from the same recruitment cohort of 208 adults, and both required complete BREQ-2 data. The analytical samples differ (n = 197 vs n = 200) only because Gruber et al. additionally required complete CFQ-R data, while the present analysis required complete PACES data. | Overlaps substantially with the present manuscript. Both studies drew from the same recruitment cohort of 208 adults and both required complete PACES data; the analytical samples differ (n = 168 vs n = 200) only because Welsner et al. additionally required complete 7-day Physical Activity Recall data, whereas the present analysis additionally required complete BREQ-2 data.  Specifically, the present sample includes 167 of the 168 participants from Welsner et al. (as stated in the main manuscript). This information has been added to ensure full consistency between the manuscript text and this table. | The three analytical samples derive from the same recruitment cohort of 208 adults at the Adult CF Centre Essen. Sample differences reflect study-specific requirements regarding questionnaire completeness. Participants with complete BREQ-2 and PACES data were retained for the present analysis (n = 200). |
| Main research question | The aim of this study was to investigate the association of Physical Activity (PA) enjoyment in adults with CF, examining the relative contributions of clinical factors, health-related quality of life (HRQoL) domains, and motivational regulation. | Does physical activity enjoyment mediate the relationship between clinical/non-clinical factors and self-reported physical activity levels? | Is pulmonary function (ppFEV1) independently associated with motivational regulation and physical activity enjoyment after accounting for CFTR modulator therapy status and sex? |
| Primary outcome(s) | PA / exercise enjoyment assessed with the Physical Activity Enjoyment Scale (PACES, German Version) [3]. | Self-reported physical activity levels by intensity, assessed with the 7-day Physical Activity Recall. | Motivational regulation assessed with BREQ-2 subscales, Relative Autonomy Index, and PA enjoyment assessed with PACES. |
| Key predictors / explanatory variables | Clinical parameters, CFQ-R health-related quality-of-life domains (assessed with the Cystic Fibrosis Questionnaire-Revised CFQ-R) [4], and BREQ-2 motivational regulation (assessed with the Behavioural Regulation in Exercise questionnaire 2. Edition, BREQ-2)) [5]. | ppFEV1, age, BMI, and physical activity enjoyment as mediator. | ppFEV1 as the clinical variable of interest; CFTR modulator therapy status and sex as adjustment variables; sensitivity analysis including age and BMI. |
| Questionnaires / instruments overlapping across studies | PACES and BREQ-2 overlap with the present manuscript; CFQ-R was additionally used in this publication. | PACES overlaps with the present manuscript; 7-day Physical Activity Recall was additionally used in this publication. | PACES and BREQ-2 are central instruments. The present analysis does not use CFQ-R domains or a validated physical activity behaviour outcome. |
| Variables already reported from the overlapping cohort | PACES was reported as the outcome; BREQ-2 subscales, RAI, CFQ-R domains, and selected clinical variables were used as predictors of PACES enjoyment. | PACES, 7-day Physical Activity Recall variables, ppFEV1, age, and BMI were reported in relation to self-reported physical activity levels and mediation by enjoyment. | Uses PACES, BREQ-2 and ppFEV₁, but analyses BREQ-2 motivational regulation subscales and RAI as outcomes in relation to ppFEV₁; CFQ-R and physical activity behaviour are not included in the final models. |
| Physical activity behaviour included? | No objective physical activity behaviour outcome; focus was on enjoyment and its predictors. | Yes. Self-reported physical activity levels were assessed with the 7-day Physical Activity Recall. | No validated physical activity behaviour outcome was included in the final models; therefore behavioural translation cannot be tested. |
| Statistical approach | Hierarchical regression models to identify predictors of PACES enjoyment. | Mediation analyses examining physical activity enjoyment as mediator between ppFEV1 and physical activity levels. | Multivariate general linear models, partial correlations, and hierarchical regressions assessing ppFEV1 associations with motivational and enjoyment outcomes. |
| Analyses novel to the present manuscript | Not applicable; this previous publication used hierarchical regression with PACES as the dependent variable. | Not applicable; this previous publication used mediation analysis with physical activity levels as dependent outcomes. | Associations between ppFEV1 and BREQ-2 motivational regulation subscales, RAI, and PACES after adjustment for CFTR modulator therapy status and sex; hierarchical regression models quantifying the incremental variance explained by ppFEV1; sensitivity analyses including age and BMI. |
| Main novel contribution | Identified intrinsic motivation as the strongest predictor of physical activity/exercise enjoyment, outweighing clinical and most HRQoL variables. | Quantified the mediating role of enjoyment in the association between ppFEV1 and vigorous / moderate-to-vigorous physical activity. | First analysis to examine whether ppFEV₁ is independently associated with the BREQ-2 motivational regulation subscales and the Relative Autonomy Index in adults with CF; extends the previous work from the same centre to the motivational regulation domain, which had not previously been examined as an outcome in relation to pulmonary function. |
| How the present manuscript differs conceptually | Previous study modelled enjoyment as the dependent variable and focused on predictors from HRQoL and motivation. | Previous study modelled physical activity behaviour as the dependent variable and enjoyment as mediator. | Current manuscript treats motivational regulation and enjoyment as outcomes and asks whether ppFEV1 is related to these psychological constructs. |
| Interpretative boundary | Does not establish whether ppFEV1 is independently associated with the full motivational regulation spectrum. | Does not establish whether ppFEV1 is associated with BREQ-2 motivational regulation subscales. | Does not test physical activity behaviour or mediation; cross-sectional associations are hypothesis-generating only. |
| Key limitation relevant to overlap | Single-centre design and overlap with later analyses from the same cohort. | Single-centre design, self-reported physical activity, and overlap with related analyses from the same cohort. | Single-centre design, overlapping dataset, small effects, residual confounding, and no validated physical activity behaviour outcome. |

**Note.** All three analytical samples derive from the same recruitment cohort of 208 adults with CF at the Adult CF Centre of the Ruhrlandklinik Essen. Sample sizes differ because of study-specific requirements regarding questionnaire completeness and availability of the variables required for each analysis.

References

1.	Gruber W, Stehling F, Schermaul J-S, Ortiz JG, Lechtenberg L, Taube C, Welsner M. Predictors of Physical Activity Enjoyment in Adults with Cystic Fibrosis: The Role of Quality of Life and Motivation-A Single-Center Study. Healthcare (Basel) 2025. doi:10.3390/healthcare13172194.

2.	Welsner M, Schermaul J-S, Ortiz JG, Lechtenberg L, Taube C, Stehling F, Gruber W. Mediating effects of physical activity enjoyment on physical activity levels in adults with cystic fibrosis. Front. Sports Act. Living. 2026;Volume 8 - 2026.

3.	Jekauc D, Voelkle M, Wagner MO, Mewes N, Woll A. Reliability, validity, and measurement invariance of the German version of the physical activity enjoyment scale. J Pediatr Psychol. 2013;38:104–15. doi:10.1093/jpepsy/jss088.

4.	Wenninger K, Aussage P, Wahn U, Staab D. The revised German Cystic Fibrosis Questionnaire: validation of a disease-specific health-related quality of life instrument. Qual Life Res. 2003;12:77–85. doi:10.1023/a:1022011704399.

5.	Witzki A, Leyk D. Behavioural Regulation in Exercise Questionnaire (BREQ-2) Reliability and Validity of a German Translation. In: Güntürkün (Hg.) 2014 – 49. Kongress der Deutschen Gesellschaft.
